# Arthrogryposis multiplex congenita—an update

**DOI:** 10.1007/s11832-015-0688-2

**Published:** 2015-10-19

**Authors:** Bjarne Møller-Madsen

**Affiliations:** Department of Children’s Orthopaedics, Aarhus University Hospital, Aarhus, Denmark

## Introduction

This special issue of *Journal of Children’s Orthopaedics* includes seven papers on arthrogryposis multiplex congenita (AMC). Arthrogryposis is derived from the Greek words arthro (joint) and gryposis (crooked). The papers are based on lectures presented during the 31st Annual Meeting of the European Paediatric Orthopaedic Society (EPOS) held in Helsinki, April 18th–21st 2012, where EPOS Educational Committee arranged a BAT Advanced Course on ‘Arthrogryposis Multiplex Congenita—an update’.

The authors present the most recent updates on the clinical features, etiology, diagnosis and management of AMC. The articles emphasize the importance of early identification and implementation of a therapy plan for an optimal outcome. AMC is a rare syndrome even though it is seen in more than 400 different clinical settings. The etiology is multifocal as approximately 200 syndromes are registered today with components that are genetically similar to AMC. Hence, the word arthrogryposis does not refer to a single syndrome but to a symptom complex, where congenital non-progressive joint contractures are the main finding. The prevalence of AMC is 1:5,100 live births; clubfoot and hip luxations are seen in 1:200 live births [1].

Today, the most widely used and accepted classification is by Bamshad et al. [2], which was published in 2009 in a review on AMC including a classification (see below courtesy F Hefti, Basel).

The management of hip pathology is comprehensively described by Christopher Bradish [3]. Open reduction through the medial approach, first described by Ludloff in 1913 [4], is recommended from the age of 3 months onwards, giving better postoperative range of motion compared to patients treated by the anterolateral approach. Eva Ponten [5] describes the management of knee joint contractures and congenital knee joint dislocation. Treatment should be initiated as early as possible, preferably within the first 24 h, primarily as manipulation and splinting for the dislocated knee. Ruth Lester [6] emphasizes the necessity of assessing the full forearm including the shoulder girdle, and not only the hand, so that no existing or potential function is diminished. The use of orthosis and gait analysis in children suffering from AMC is fully described by Åsa Bartonek [7]. Finally, Jean Dubousset and Michel Guillaumat [8] describe the long-term outcome for patients with AMC. Sixty-five patients were reviewed and no loss of function beyond adolescence was reported.

The papers highlight the challenge that AMC poses to every physician. In order to give the best management to each child, a thorough examination by a team of dedicated physicians with a wide range of up-to-date skills and knowledge is recommended in order to benefit the children suffering from AMC.

## Classification of arthrogryposis (from: F Hefti, Pediatric Orthopaedics in Practice, 2nd edition, Springer 2015)

### Amyoplasia

This is primarily a muscle disorder that affects all four extremities, usually symmetrically. The shoulders are usually internally rotated and adducted, the elbows are overstretched, the wrists flexed, the hips dislocated, the knees overstretched and the feet in a pronounced clubfoot position. 10 % of the patients also have abdominal involvement. Occasionally, the extremities are affected asymmetrically, or (in rare isolated cases) only the upper extremities are damaged. No hereditary component has been established for amyoplasia.

### Distal arthrogryposis

This is a group of autosomal-dominant inherited diseases that primarily affect the distal parts of the extremities. Ten subgroups are known, at least five of which are triggered by various genes. Characteristic features of the more common types include the following:

- *Type* *1* This type is characterized by camptodactyly and clubfeet. The interdigital folds are often missing.
- *Type* *2* Phenotypically, this form corresponds to the Freeman−Sheldon syndrome. In addition to contractures of hands and feet (as in type 1), oropharyngeal anomalies, scolioses and facial changes (narrow mouth, puckered lips and an H-shaped dimple of the chin) also occur.
- *Type* *3* This type is also known as Gordon syndrome and is associated with stunted growth and cleft palate.
- *Type* *5* As well as contractures, this type also involves ocular anomalies (ptosis, restricted eye movements, strabismus).
- *Type* *7* This type is associated with stunted growth.
- *Type* *9* This corresponds to the contractural form of arachnodacytly and was formerly considered to be combined with Marfan syndrome.

### Arthrogryposes with CNS etiology

Developmental disorders affecting the frontal lobe (hydranencephaly, microcephaly) are occasionally associated with arthrogryposes. Accordingly, the contractures are attributed to a reduced cortical activation of motor neurons.

### Neuromuscular causes of arthrogryposis

Genetic peripheral neuropathies have been described, but are very rare causes of arthrogryposes. Mothers with myasthenia gravis and autoantibodies that target fetal acetylcholine receptors can present this type of arthrogryposis. In theory, the repeated administration of botulinum toxin to pregnant women may also trigger such a reaction, although this etiology has not been observed to date in children with arthrogryposis. Congenital myopathies may also be associated with an arthrogryposis.
